# Patient Perspective on Preoperative Simulation in Plastic Surgery: A Systematic Review of Patient-Reported Outcomes

**DOI:** 10.1177/22925503261478090

**Published:** 2026-08-31

**Authors:** Omar El Sewify, Andrew Gorgy, Marion Sylvain, Jack Legler, Dino Zammit

**Affiliations:** 1 12369 Faculty of Medicine, Laval University, Québec City, Quebec, Canada; 2 Division of Plastic and Reconstructive Surgery, 5620 McGill University Health Center, Montreal, Quebec, Canada; 3 Faculty of Medicine and Health Sciences, 12367 McGill University, Montreal, Quebec, Canada; 4 Division of Orthopaedic Surgery, 5620 McGill University Health Center, Montreal, Quebec, Canada

**Keywords:** simulation, surgery, satisfaction, aesthetic, simulation, chirurgie, satisfaction, esthétique

## Abstract

**Introduction:**

Preoperative simulation technologies are increasingly utilized in plastic surgery to enhance surgical planning and patient communication. While their technical benefits have been established, the impact on patient-reported outcomes (PROs), in particular satisfaction and perceived accuracy, remain underexplored. This systematic review evaluated the effect of preoperative simulation on patient-reported satisfaction, decision-making, and perceived accuracy in breast and facial aesthetic plastic surgery.

**Methods:**

A systematic search of MEDLINE, Embase, and Cochrane databases up to April 2025 identified studies reporting PROs in adult patients undergoing breast (aesthetic or reconstructive) or facial aesthetic surgery with preoperative simulation. Data on demographics, simulation type, satisfaction, accuracy, and complications were extracted and analyzed.

**Results:**

Seventeen studies (1333 patients; 970 simulation, 363 control) met inclusion criteria. Breast surgery patients using simulation showed higher satisfaction with appearance (Breast-Q: 75.0 vs 58.1), surgical outcomes (84.9% vs 78.8%), and psychosocial well-being. Simulation patients reported higher satisfaction with the simulation experience (95.4%) and greater trust in their surgeon (95.2%), with lower complication rates (4.1% vs 41.5%). In facial surgery, simulation users demonstrated greater overall satisfaction (79.2% vs 67.3%) and improved understanding of outcomes. Perceived accuracy was higher in breast (91.0%) than facial surgery (74.1%). Across procedures, simulation enhanced shared decision-making and surgeon-patient rapport.

**Conclusion:**

Current evidence suggests that preoperative simulation may enhance patient satisfaction, trust, and engagement in plastic surgery, particularly in breast procedures. However, findings are based on heterogeneous studies with diverse patient populations, simulation technologies, and outcome measures. Further prospective comparative studies using standardized PRO measures are needed to better define the role of preoperative simulation across aesthetic and reconstructive plastic surgery.

## Résumé

**Introduction:**

Les technologies de simulation préopératoire sont de plus en plus utilisées en chirurgie plastique pour améliorer la planification de l’intervention et la communication avec les patients. Leurs avantages techniques sont bien établis, mais leurs effets sur les résultats déclarés par les patients (RDP), notamment la satisfaction et la perception de précision, demeurent peu étudiés. La présente analyse systématique a permis d’évaluer l’effet de la simulation préopératoire sur la satisfaction déclarée par les patients, le processus décisionnel et la perception de précision en chirurgie esthétique mammaire et faciale.

**Méthodes:**

Après une fouille systématique des bases de données MEDLINE, Embase et Cochrane jusqu’en avril 2025, les chercheurs ont recensé les études abordant les résultats déclarés par des patients adultes ayant subi une chirurgie mammaire (esthétique ou reconstructive) ou une chirurgie esthétique faciale conjuguées à une simulation préopératoire. Ils ont extrait et analysé les données sur les caractéristiques démographiques, le type de simulation, la satisfaction, la précision et les complications.

**Résultats:**

Au total, 17 études (1 333 patients; 970 incluant la simulation, 363 sujets-témoins) respectaient les critères d’inclusion. Les patientes qui avaient subi une chirurgie mammaire après une simulation étaient plus satisfaites de leur apparence (Breast-Q : 75,0 par rapport à 58,1), de leurs résultats chirurgicaux (84,9 % par rapport à 78,8 %) et de leur bien-être psychosocial. Celles qui avaient été exposées à la simulation étaient satisfaites de l’expérience de simulation (95,4 %), avaient plus confiance en leur chirurgien (95,2 %) et présentaient des taux de complications plus faibles (4,1 % par rapport à 41,5 %). En chirurgie faciale, les personnes exposées à la simulation présentaient une meilleure satisfaction globale (79,2 % par rapport à 67,3 %) et comprenaient mieux les résultats attendus. La perception de précision était plus élevée en chirurgie mammaire (91,0 %) qu’en chirurgie faciale (74,1 %). Toutes interventions confondues, la simulation a favorisé des décisions communes et la relation entre le chirurgien et les patients.

**Conclusion:**

Selon les données actuelles, la simulation préopératoire pourrait améliorer la satisfaction, la confiance et la participation des patients en chirurgie plastique, particulièrement dans le cadre d’interventions mammaires. Ces résultats reposent toutefois sur des études hétérogènes aux populations de patients, aux technologies de simulation et aux mesures de résultats diversifiés. D’autres études prospectives comparatives faisant appel à des mesures normalisées des RDP devront être réalisées pour mieux définir le rôle de la simulation préopératoire en chirurgie esthétique et reconstructive.

## Introduction

Preoperative simulation technologies have transformed surgical planning in aesthetic and reconstructive surgery. Tools such as 3D surface imaging and virtual simulation platforms allow patients to visualize anticipated outcomes before surgery.^1^ These innovations are increasingly used in breast surgery, particularly for oncologic reconstruction and cosmetic augmentation, and are gaining adoption in facial aesthetic procedures, especially rhinoplasty surgery. However, most studies have focused on technical accuracy, surgeon utility, and training applications,^2‐5^ while the impact on the patient's subjective experience, including satisfaction and perceived involvement in decision-making, requires further investigation.

Patient-reported outcomes (PROs) have become a cornerstone for evaluating success in aesthetic surgery.^6^ Tools such as BREAST-Q and FACE-Q are widely accepted standards for assessing patient satisfaction, quality of life, and psychosocial well-being after aesthetic procedures.^1^ In a field where outcomes are not solely judged by clinical metrics but also by personal satisfaction and quality of life, tools that facilitate preoperative communication and expectation management are particularly valuable.^7^ Preoperative simulation may enhance patient understanding, facilitate shared decision-making, and improve satisfaction with outcomes.^8^ Yet, its effects on broader patient-centered outcomes, including the decision-making experience, perceived usefulness, and postoperative satisfaction, remain insufficiently studied.

In breast surgery, especially oncologic reconstruction where patients face significant psychological and physical transitions, simulation may provide reassurance.^1^ In aesthetic breast and facial procedures, where expectations strongly influence satisfaction, simulation can help clarify goals and improve surgeon-patient alignment.^8‐10^ Despite widespread use of systems like Vectra XT and Crisalix, few studies have assessed their impact on outcomes reported directly by patients.^11‐13^

This systematic review evaluates the impact of simulation technology on PROs in plastic surgery, focusing on aesthetic breast and facial procedures. By synthesizing evidence on satisfaction, perceived accuracy, and shared decision-making, it aims to clarify how simulation influences the patient experience in aesthetic and reconstructive surgery.

## Materials and Methods

This systematic review was conducted in accordance with the 2020 Preferred Reporting Items for Systematic Reviews and Meta-Analyses guidelines.^14^ The population of interest included adult patients undergoing breast surgery and facial surgery who were exposed to preoperative simulation technologies as part of surgical planning.

### Search Strategy

A comprehensive search of MEDLINE (via Ovid), Embase (via Ovid), and Cochrane Library was conducted from inception to April 2025. Citations were managed in EndNoteTM X9, and after deduplication were imported into Covidence for screening. Title/abstract and full-text screening were performed independently by 2 reviewers (OE, JL), with disagreements resolved by consensus or discussion with a third reviewer (AG).

### Study Eligibility

Strict inclusion and exclusion criteria were established. Eligible study designs included randomized controlled trials, prospective and retrospective studies, case–control studies, case series, and case reports. Systematic reviews, meta-analyses, letters, commentaries, cadaveric studies, incomplete abstracts, and non-English studies were excluded. Studies with or without comparator groups were eligible. Adult patients (≥18 years) undergoing breast or facial plastic surgery with preoperative simulation as part of surgical planning were included, while pediatric patients were excluded. Studies were required to evaluate outcomes reported directly by patients and include terms related to simulation, artificial intelligence, or plastic surgery.

### Data Extraction and Synthesis

Data extraction was performed by 2 reviewers, with disagreements resolved by consensus with a third reviewer. Extracted data included study characteristics (authors, year, design, funding, country, and study purpose), patient demographics (sample size, sex, age, and procedure type), simulation characteristics (technology used and simulation time), and outcomes (PROs, accuracy, complications, and follow-up duration). Data are summarized in comparative and descriptive tables (Supplemental Tables S1-S4).[Table table2-22925503261478090][Table table3-22925503261478090][Table table4-22925503261478090]

**Table 1.** Summary of Patient Characteristics.

General Characteristics	Total			Breast Surgery			Facial Surgery		
	Total	Sim	Control	Total	Sim	Control	Total	Sim	Control
# of patients	1333	970	363	606	453	153	727	517	210
Mean age, years	35.46 (n = 1102)	33.29 (n = 637)	55.58 (n = 121)	43.93 (n = 456)	36.53 (n = 266)	55.58 (n = 121)	29.49 (n = 646)	30.96 (n = 371)	NR
Sex (%)	M: 275 (21.5) F: 1002 (78.5)	NR	NR	M: 0 (0) F: 606 (100)	NR	NR	M: 275 (41.0) F: 396 (59.0)	NR	NR
BMI, kg/m^2^	25.93 (n = 318)	24.34 (n = 156)	27.66 (n = 93)	25.93 (n = 318)	24.34 (n = 156)	27.66 (n = 93)	NR	NR	NR
Follow-up in months	12.15 (n = 961)	9.64 (n = 431)	6.6 (n = 13)	8.8 (n = 552)	8.37 (n = 353)	6.6 (n = 13)	16.67 (n = 409)	15.40 (n = 78)	NR

Abbreviations: BMI, body mass index; F, female; M, male; NR, not reported; Sim, simulation.

**Table 2. table2-22925503261478090:** Breast-Q Outcomes.

	Simulation Group		Control Group	
Breast-Q Domain (%)	Before Surgery (n = 109)	After Surgery (n = 135)	Before Surgery (n = 45)	After Surgery (n = 73)
Overall satisfaction with breast	35.1 (SD 18.25)	75.0 (SD 16.65)	49.6 (SD 18.71)	58.1 (SD 16.97)
Change in Q-score	39.9 (SE 2.26)		8.5 (SE 3.43)	
Satisfaction with surgical outcome	NA	84.9 (SD 14.85)	NA	78.8 (SD 19.07)
Psychosocial well-being	52.7 (SD 18.61)	84.8 (SD 15.20)	59.2 (SD 17.31)	82.9 (SD 16.88)
Change in Q-score	32.1 (SE 2.21)		23.7 (SE 3.25)	
Sexual well-being	44.3 (SD 18.67)	81.1 (SD 16.61)	55.7 (SD 19.10)	78.0 (SD 19.07)
Change in Q-score	36.8 (SE 2.29)		22.3 (SE 3.62)	
Physical well-being	72.2 (SD 17.80)	75.2 (SD 12.27)	69.9 (SD 17.50)	76.0 (SD 16.27)
Change in Q-score	3.0 (SE 2.01)		6.1 (SE 3.23)	
Satisfaction with information	NA	85.2 (SD 17.90)	NA	77.1 (SD 12.79)
Satisfaction with surgeon	NA	96.6 (SD 6.50)	NA	93.7 (SD 11.03)
Satisfaction with medical staff	NA	97.6 (SD 8.24)	NA	97.6 (SD 8.27)
Satisfaction with office staff	NA	97.9 (SD 5.19)	NA	98.1 (SD 7.41)

Abbreviations: n, number of patients; NA, not applicable; SD, standard deviation; SE, standard error.

**Table 3. table3-22925503261478090:** Surgical Outcomes in Breast Surgery.

Outcome (%)	Simulation Group	Control Group
Overall satisfaction with surgical outcomes	92.5 (SD 13.25) (n = 150)	81.8 (SD 13.4) (n = 32)
Satisfaction with breast size		
Satisfied	83.7 (n = 242/289)	75.0 (n = 21/28)
Wants larger	20.8 (n = 37/178)	NR
Wants smaller	1.1 (n = 2/178)	NR
Satisfaction with breast shape	90.9 (n = 30/33)	78.6 (n = 22/28)
Satisfaction with simulation	95.4 (n = 62/65)	NA
Perceived value of simulation		NA
Necessary/very important	44.0 (n = 99/225)	
Useful/important	27.87 (n = 52/188)	
Unnecessary/not so important	6.9 (n = 13/188)	
Patient preference		NA
Very much	76.6 (n = 144/188)	
Reasonably or very much	86.2 (n = 162/188)	
Little or not needed	13.8 (n = 26/188)	
Choosing prosthesis		NA
A lot	77.1 (n = 145/188)	
A lot/Yes	96.3 (n = 181/188)	
A little/No	3.7 (n = 7/188)	
Choosing surgeon		NA
Yes	92.5 (n = 37/40)	
Not really/No	7.5 (n = 3/40)	
Improved trust in surgeon	95.2 (n = 60/63)	91.7 (n = 55/60)
Accuracy of simulation	91.0 (n = 273/300)	71.6 (n = 20/28)
Complication	4.1 (n = 14/341)	41.5 (n = 17/41)

Abbreviations: n, number of patients; NA, not available; NR, not reported; SD, standard deviation.

**Table 4. table4-22925503261478090:** Surgical Outcomes in Facial Surgery.

Outcome (%)	Simulation Group	Control Group
Overall satisfaction with surgical outcomes	79.2 (n = 122/154)	67.3 (n = 105/156)
Satisfaction with nose shape	100 (n = 24/24)	NR
Satisfaction with simulation		
Before surgery	96.3 (n = 26/27)	81.8 (n = 18/22)
After surgery	96.4 (n = 27/28)	92.9 (n = 26/28)
Perceived value of simulation^a^	68.3 (n = 112/164)	NA
Patient preference^b^	68.3 (n = 112/164)	NA
Confidence to undergo surgery from simulation^c^	70.9 (n = 273/385)	NA
Doctor-patient relationship		NA
Expressing wishes	84.0 (n = 63/75)	
Enhanced relationship	82.0 (n = 41/50)	
Modifications^d^	52.0 (n = 39/75)	NA
Changes^e^	79.5 (n = 31/39)	
Improved trust in surgeon	46.7 (n = 35/75)	NA
Accuracy of simulation	74.1 (n = 106/143)	NA
Complication	0.0 (n = 0/28)	NR

Abbreviations: n, number of patients; NA, not available; NR, not reported.

a 3D simulation helped them better understand the deformity of their nose and the aim of the surgery than 2D simulation

b 3D simulation is more attractive and innovative than 2D simulation

c 3D simulation made them feel more assured to undergo the surgery than 2D simulation/Easier to commit to the procedure

d Modifications suggested during the imaging that they had not previously considered

e Adopted the changes suggested during imaging

**Table 5.** Comparison of Simulation Outcomes Across Breast and Facial Patients.

Outcome (%)	Breast Surgery	Facial Surgery
Overall satisfaction with surgical outcomes	92.5 (n = 139/150)	79.2 (n = 122/154)
Satisfaction with breast/nose shape	90.9 (n = 30/33)	100 (n = 24/24)
Satisfaction with simulation	95.4 (n = 62/65)	96.4 (n = 27/28)
Perceived value of simulation	72.8 (n = 156/214)	68.3 (n = 112/164)
Patient preference	86.2 (n = 162/188)	68.3 (n = 112/164)
Improved trust in surgeon	95.2 (n = 60/63)	46.7 (n = 35/75)
Accuracy of simulation	91.0 (n = 273/300)	74.1 (n = 106/143)
Complication	4.1 (n = 14/341)	0.0 (n = 0/28)

Abbreviation: n, number of patients.

### Statistical Analysis

Data points were pooled using means and weighted averages where applicable. Outcomes of interest included patient-reported satisfaction with simulation, satisfaction with surgical outcomes (eg, shape, size), perceived accuracy of the simulation, and its value in expectation-setting or shared decision-making. Given the substantial clinical and methodological heterogeneity among included studies, including differences in patient populations, surgical procedures, simulation platforms, study designs, and PRO measures (PROMs), formal meta-analysis was not feasible. Consequently, data were synthesized descriptively, and no inferential comparisons were performed across studies.

## Results

### Study Characteristics

**Figure 1.**
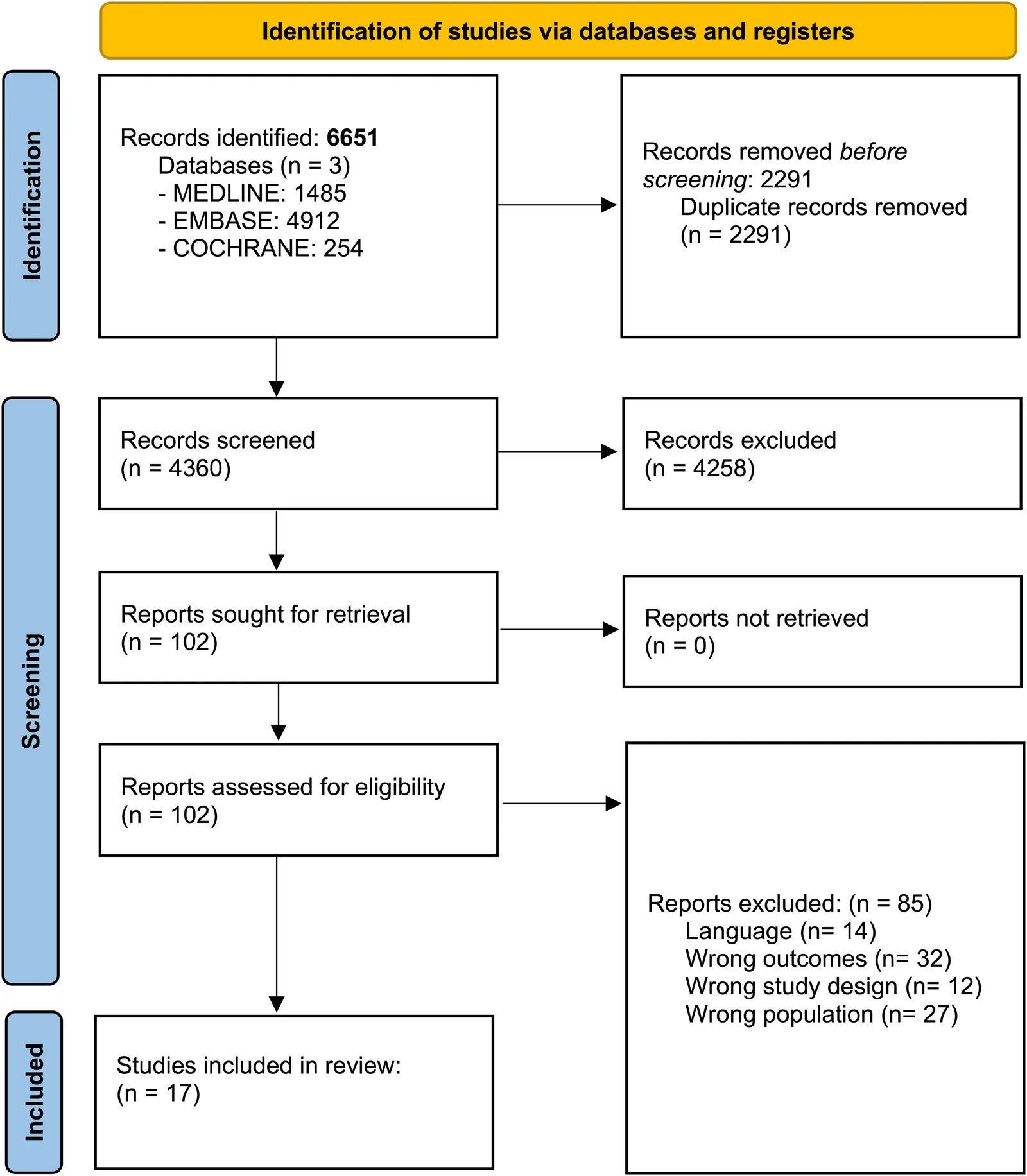
PRISMA flow diagram.

The initial search identified 6651 articles. After removal of 2291 duplicates, 4360 titles and abstracts were screened. Of these, 102 articles underwent full-text review, and 17 met the inclusion criteria (Figure 1; Supplemental Table S1).

### Patient Demographics

A total of 1333 patients were included, with 970 patients in the simulation group and 363 in the control group. Of these, 606 patients underwent breast surgery and 727 underwent facial aesthetic surgery. Within the breast surgery subgroup, 453 patients were exposed to preoperative simulation, while 153 served as controls. In the facial surgery subgroup, predominantly rhinoplasty procedures, 517 patients were in the simulation group and 210 in the control group (Table 1).

The mean age across all patients was 35.46 years (n = 1102). Breast surgery patients were older (mean 43.93 years, n = 456), compared to facial surgery patients (mean 29.49 years, n = 646). Within the breast surgery subgroup, the mean age was 36.53 years in the simulation group and 55.58 years in the control group. In the craniofacial group, the simulation cohort had a mean age of 30.96 years, while age was not reported for controls (Table 1).

Sex was reported for 1277 patients, with the cohort predominantly female (78.5%). All breast surgery patients were female, while the craniofacial surgery group included both sexes (Table 1).

BMI was reported for 318 patients (mean of 25.93 kg/m^2^). The simulation group had a slightly lower mean BMI than controls (24.34 kg/m^2^ vs 27.66 kg/m^2^). BMI was only reported in breast surgery studies (Table 1).

The mean follow-up across studies was 12.15 months (n = 961), longer for craniofacial patients (16.67 months, n = 409) than breast surgery patients (8.8 months, n = 552). Follow-up averaged 9.64 months in the breast simulation group versus 6.6 months in controls. In the craniofacial simulation subgroup, follow-up averaged 15.4 months; control follow-up was not reported (Table 1).

### Simulation Characteristics

Procedures included both aesthetic and reconstructive plastic surgery, primarily implant-based augmentation, breast-conserving surgery, mastectomy with implant-based reconstruction, and aesthetic or functional rhinoplasty. A limited number of studies also examined craniofacial procedures such as otoplasty and chin augmentation, though these were not included in the analysis per the study criteria.

A variety of preoperative simulation technologies were utilized, including 3D imaging platforms such as Vectra (M3, XT, and H1 models), Crisalix, and Arbrea Breast Software. Some studies employed 2D image manipulation tools like Photoshop CS3, while others integrated more advanced systems such as Morpheus Photo Warper, RhinOnBlender, and DreamWizard. In some protocols, simulations were complemented by 3D-printed models such as the Zortrax^®^ printer. Reported simulation time ranged from 90 s to over 2 h (Supplemental Table S3).

### Breast Surgery Outcomes

Across breast surgery studies, patients who underwent preoperative simulation reported higher satisfaction and improved PROs compared to controls.

Breast-Q scores showed marked improvements in the simulation group, with satisfaction with breast appearance increasing from 35.1 (SD 18.25) preoperatively to 75.0 (SD 16.65) postoperatively, compared to an increase from 49.6 (SD 18.71) to 58.1 (SD 16.97) in controls. Although both groups showed improvement, postoperative scores were higher in the simulation group. Simulation patients also reported greater satisfaction with surgical outcomes (84.9 vs 78.8) and greater improvements in psychosocial and sexual well-being, while physical well-being was similar between groups (Table 2).

Overall satisfaction with surgical outcomes was higher in the simulation group (92.5% vs 81.8%). Satisfaction with breast size was reported by 83.7% of simulation patients versus 75.0% of controls, and satisfaction with breast shape was also higher in the simulation group (90.9% vs 78.9%) (Table 3).

Satisfaction with the simulation experience was very high, with 95.4% of patients reporting satisfaction. Nearly three-quarters of patients perceived simulation as valuable, with 44.0% rating it “necessary” or “very important” and 27.9% “useful” or “important.” Additionally, 76.6% reported liking the experience “very much,” and 86.2% found it helpful in preparing for surgery (Table 3).

Simulation also influenced decision-making. 77.1% reported it helped in choosing their prosthesis and 96.3% reported at least some influence. Furthermore, 92.5% stated simulation influenced their choice of surgeon and 95.2% reported increased trust in their surgeon (Table 3).

Perceived accuracy was higher with simulation, with 91.0% reporting that surgical outcomes matched simulated images compared with 71.6% in controls. Complication rates were also lower in the simulation group (4.1%, 14/341) compared to controls (41.5%, 17/41) (Table 3).

### Facial Surgery Outcomes

In facial aesthetic surgery, primarily rhinoplasty, preoperative simulation was associated with higher patient-reported satisfaction and improved engagement during the preoperative consultations.

Overall satisfaction with surgical outcomes was higher in the simulation group compared with controls (79.2% vs 67.3%). Among patients reporting satisfaction with nose shape, 100% of those in the simulation group (n = 24) were satisfied, although no comparative control data were available (Table 4).

Satisfaction with the simulation experience was consistently high. In the simulation group, 96.3% of patients reported satisfaction before surgery and 96.4% after surgery, compared with 81.8% and 92.9% in the control group, respectively (Table 4).

Patients also reported high perceived value of simulation. Among simulation users, 68.3% found it helpful in understanding their nasal deformity and expected outcomes, and the same proportion preferred 3D simulation over 2D alternatives. Additionally, 70.9% reported that simulation increased their confidence in proceeding with surgery (Table 4).

Simulation also improved aspects of the surgeon-patient relationship. Most patients reported that it helped them better express aesthetic goals (84.0%) and improved their relationship with the surgeon (82.0%). Additionally, 46.7% reported increased trust in their surgeon (Table 4).

Simulation influenced decision-making as well. 52.0% of patients reported being offered modifications during the simulation session that they had not previously considered, and 79.5% adopted these changes in the final surgical plan. Perceived accuracy was also high, with 74.1% reporting that surgical outcomes matched simulated images. No complications were reported in the simulation group (0%, n = 0/28), though control complication data were unavailable (Table 4).

### Comparative Outcomes Between Breast and Facial Surgery Patients

When comparing breast and facial aesthetic surgery patients, several differences in PROs and perceptions of simulation emerged.

Overall satisfaction with surgical outcomes was higher among breast surgery patients (92.5%, n = 139/150) than facial surgery patients (79.2%, n = 122/154). Satisfaction with the operated area shape was high in both groups (90.9% vs 100%) (Table 5).

Satisfaction with the simulation experience was similarly high in both groups (95.4% vs 96.4%), and the perceived value of simulation was comparable (72.8% vs 68.3%) (Table 5). However, preference for simulation was greater among breast surgery patients (86.2%, n = 162/188) compared with facial surgery patients (68.3%, n = 112/164).

Trust-related outcomes differed between groups. In breast surgery studies, 95.2% of patients reported trusting their surgeon, whereas facial surgery studies evaluated whether simulation increased trust, with 46.7% reporting improvement. These measures are not directly comparable due to differences in survey questions (Table 5).

Perceived simulation accuracy was higher in breast surgery patients (91.0%, n = 273/300) than in facial surgery patients (74.1%, n = 106/143). Reported complication rates were 4.1% in breast surgery (n = 14/341) and 0% in facial surgery (n = 0/28) (Table 5).

### Risk of Bias Analysis

Risk of bias was evaluated using the Cochrane Risk of Bias 2 tool for randomized controlled trials and the Methodological Index for Non-Randomized Studies for observational studies. Both randomized trials demonstrated low risk of bias. Among comparative observational studies, scores ranged from 10 to 18 out of 24, reflecting moderate to good methodological quality. Noncomparative studies scored between 9 and 13 out of 16. Overall, while randomized studies showed low risk of bias, observational studies demonstrated variable methodological rigor, with some limitations in study design and reporting (Supplemental Table S4).

## Discussion

### Summary of Findings

This systematic review assessed the impact of preoperative simulation on PROs in aesthetic and reconstructive plastic surgery, focusing on both breast and facial procedures. Across 17 studies and 1333 patients, simulation technologies such as 3D surface imaging and virtual morphing tools were associated with improved patient satisfaction, enhanced decision-making, and better patient-surgeon communication. Overall, simulation demonstrated high satisfaction rates, strong perceived accuracy, and a meaningful influence on the patient experience.

Across both breast and facial surgery cohorts, patients reported high satisfaction with simulation and postoperative outcomes. In breast surgery, simulation was associated with higher satisfaction with breast shape and size, greater trust in the surgeon, and improved overall outcomes. Facial surgery patients also reported high satisfaction, particularly in visualizing expected changes and communicating surgical goals, though perceived accuracy and preference for simulation were higher in breast surgery patients.

### Simulation Enhances Patient Satisfaction and Engagement

Our findings align with existing literature demonstrating that visual simulation technologies improve communication and patient experience in aesthetic surgery. Platforms such as Vectra XT and Crisalix allow patients to visualize expected results, reducing preoperative anxiety and facilitating informed consent.^15‐17^ Studies have reported high satisfaction with simulation tools, including Arbrea Breast Software (VAS 8.2/10) and improved understanding of surgical procedures in randomized trials of breast reconstruction (*P* = .021).^9,18^ Portable systems like Crisalix have shown good predictive value in selected cases.^8^

In our review, both breast and facial surgery patients reported high satisfaction with the simulation experience (<95%), highlighting its role in improving preoperative preparation. Breast surgery patients, however, reported higher satisfaction with outcomes and greater perceived accuracy, suggesting simulation may have greater impact in body contouring and reconstruction.

### Contextualizing the Breast Versus Facial Surgery Differences

Breast surgery patients were more likely to report trust in their surgeon, stronger preference for simulation, and greater perceived accuracy of simulated outcomes. One explanation may lie in the psychological context of breast surgery, where patients may experience changes in body image, identity, or femininity. In these situations, visual simulation tools can provide reassurance and help restore confidence and autonomy.^8,19,20^

By contrast, facial aesthetic procedures, especially rhinoplasty, are often pursued for refinement rather than restoration. Patients seeking facial surgery have been reported to exhibit higher rates of body image dissatisfaction, perfectionistic expectations, and psychological vulnerabilities such as body dysmorphic disorder.^21,22^ While simulation may help set realistic expectations, its ability to address deeper aesthetic dissatisfaction may be limited. Interestingly, 46.7% of facial surgery patients reported increased trust due to simulation, whereas 95.2% of breast patients reported trusting their surgeon. However, these findings are difficult to compare directly because the surveys measured different constructs (overall trust vs change in trust), highlighting the need for standardized PROMs.

### Alignment with Prior Research

The value of preoperative simulation in rhinoplasty is supported by several studies. Lekakis et al reported that 82.5% of postoperative results were rated as better than the simulations by a blinded panel (*P* < .001), reinforcing simulation's role in communication while acknowledging its predictive limitations.^23^ Other studies comparing 3D morphing to 2D imaging found that 95% of patients, particularly women and those undergoing revision procedures, considered 3D simulation more valuable than traditional methods.^24^ Quantitative assessments have similarly shown that postoperative outcomes are often judged aesthetically superior to simulations, reinforcing simulation's primary value as a communication tool rather than a strict prediction.^25^

In breast reconstruction, a randomized trial showed that 3D simulation improved satisfaction with preoperative information (*P* = .021), with 86.6% of oncoplastic patients reporting better understanding of their procedure.^9^ A multicenter study also found that satisfaction with preoperative information and surgeon interaction strongly predicted postreconstruction satisfaction (information *P* < .001; surgeon *P* < .001).^7^ Together, these findings support the results of this review, demonstrating measurable benefits of simulation in both facial and breast aesthetic surgery.

### Implications for Clinical Practice

Simulation tools should be viewed not just as planning aids but as instruments of patient-centered care. Evidence shows that 3D models foster clearer understanding, informed consent, and shared decision-making. For rhinoplasty, simulation allows patients to visualize potential outcomes, reducing decisional uncertainty and helping surgeons address aesthetic expectations. Interactive visualization of different nasal shapes may also reduce anxiety and, in some practices, has been associated with lower revision rates, suggesting potential cost savings.

In breast reconstruction, visual feedback has been linked to greater satisfaction with preoperative information and overall care, factors closely associated with patient well-being and outcomes.^9^ Beyond predicting morphology, simulation can provide reassurance, strengthen trust, and improve patient-surgeon rapport in emotionally significant aesthetic procedures.

Surgeons should consider incorporating simulation into preoperative consultations, particularly when managing complex expectations. However, costs, learning curves, and limited accessibility may restrict widespread adoption, and clinics should balance these factors when integrating simulation technologies into practice.

### Limitations and Future Directions

This review is limited by substantial clinical and methodological heterogeneity across the included studies, including differences in patient populations, surgical procedures (eg, breast augmentation, breast reconstruction, breast-conserving surgery, and rhinoplasty), simulation technologies (2D imaging, 3D surface imaging, augmented reality, and 3D-printed models), study designs, and outcome measures. Many studies used nonstandardized or modified PROMs, including variations in the BREAST-Q, limiting comparability and precluding formal meta-analysis. Variability in questionnaire design and wording, particularly for constructs such as trust, satisfaction, and shared decision-making, further complicated comparisons across studies. Consequently, findings were synthesized descriptively and should be interpreted with appropriate caution.

Key statistical information, such as standard deviations and complete outcome reporting, was frequently missing, limiting the ability to quantitatively compare findings across studies and precluding meta-analysis for most outcomes. Several studies also included patients undergoing secondary procedures (eg, revision rhinoplasty), which may have influenced expectations and satisfaction but were not consistently distinguished.

Protocol transparency was limited, with few studies reporting when simulations were performed or how they informed final surgical planning. Additionally, follow-up periods were often shorter in control groups, restricting comparisons of long-term outcomes such as satisfaction and complications.

## Conclusion

Current evidence suggests that preoperative simulation may improve patient satisfaction, communication, and shared decision-making in aesthetic and reconstructive plastic surgery. Across the included studies, patients generally reported high satisfaction with the simulation experience, perceived simulation as a valuable aid during the consultation process, and described improved understanding of expected surgical outcomes. These findings appeared particularly consistent in breast surgery, although encouraging results were also reported in facial aesthetic procedures.

However, the available evidence is limited by substantial heterogeneity in simulation technologies, study designs, and PROM, which precluded formal quantitative synthesis. Consequently, these findings should be interpreted with care. Additional prospective comparative studies using validated PROM are needed to better define the clinical impact of preoperative simulation and determine the settings in which it provides the greatest benefit.

## Supplemental Material

sj-docx-1-psg-10.1177_22925503261478090 - Supplemental material for Patient Perspective on Preoperative Simulation in Plastic Surgery: A Systematic Review of Patient-Reported Outcomes Supplemental material, sj-docx-1-psg-10.1177_22925503261478090 for Patient Perspective on Preoperative Simulation in Plastic Surgery: A Systematic Review of Patient-Reported Outcomes by Omar El Sewify, Andrew Gorgy, Marion Sylvain, Jack Legler and Dino Zammit in Plastic Surgery

sj-docx-2-psg-10.1177_22925503261478090 - Supplemental material for Patient Perspective on Preoperative Simulation in Plastic Surgery: A Systematic Review of Patient-Reported Outcomes Supplemental material, sj-docx-2-psg-10.1177_22925503261478090 for Patient Perspective on Preoperative Simulation in Plastic Surgery: A Systematic Review of Patient-Reported Outcomes by Omar El Sewify, Andrew Gorgy, Marion Sylvain, Jack Legler and Dino Zammit in Plastic Surgery

sj-docx-3-psg-10.1177_22925503261478090 - Supplemental material for Patient Perspective on Preoperative Simulation in Plastic Surgery: A Systematic Review of Patient-Reported Outcomes Supplemental material, sj-docx-3-psg-10.1177_22925503261478090 for Patient Perspective on Preoperative Simulation in Plastic Surgery: A Systematic Review of Patient-Reported Outcomes by Omar El Sewify, Andrew Gorgy, Marion Sylvain, Jack Legler and Dino Zammit in Plastic Surgery

sj-docx-4-psg-10.1177_22925503261478090 - Supplemental material for Patient Perspective on Preoperative Simulation in Plastic Surgery: A Systematic Review of Patient-Reported Outcomes Supplemental material, sj-docx-4-psg-10.1177_22925503261478090 for Patient Perspective on Preoperative Simulation in Plastic Surgery: A Systematic Review of Patient-Reported Outcomes by Omar El Sewify, Andrew Gorgy, Marion Sylvain, Jack Legler and Dino Zammit in Plastic Surgery
