# Supplementary material for: Patient Perspective on Preoperative Simulation in Plastic Surgery: A Systematic Review of Patient-Reported Outcomes
Source: Plast Surg (Oakv). 2026 Aug 31:22925503261478090. Online ahead of print. doi: 10.1177/22925503261478090 (PMC13530028; doi:10.1177/22925503261478090)
Supplement: Supplementary file 1 [file sj-docx-1-psg-10.1177_22925503261478090.docx]

**Supplemental Table 1. Study Characteristics**

| Author | Year | Country | Funding | Methodology | Purpose/Aim |
| --- | --- | --- | --- | --- | --- |
| Costa et al. | 2017 | USA | None | Prospective cohort | 1) To utilize 3D imaging to determine if preoperative bra sizing provides equivocal information compared to surgical simulation for patient education prior to a breast augmentation. |
| Creasman et al. | 2011 | USA | None | Prospective cohort | Describe the application of four-dimensional technology during patient consultation to assist in planning implant size and type. |
| de Runz et al. | 2017 | France | NR | Retrospective cohort | 1) To assess the patients’ attitudes regarding 3D simulation for breast augmentation  2) To evaluate the subsequent benefits. |
| Donfrancesco et al. | 2013 | Sweden | NR | Prospective cohort | Investigate patient satisfaction and correlation between prediction and actual outcome using 3D simulated images in breast augmentation |
| Godden et al. | 2021 | UK | Yes | Randomized controlled trial | 1) To establish which preoperative intervention best prepares women for their likely aesthetic outcome after BCS: verbal description alone; viewing others’ (2D) photographs; or viewing a 3D simulation of their own likely appearance. |
| Hutchinson et al. | 2021 | USA | None | Retrospective cohort | 1) To determine if preoperative 3D imaging improves the breast augmentation consultation. |
| Kuroda et al. | 2024 | Brazil | None | Randomized controlled trial | 1) To evaluate preoperative patient expectations and the impact of 3D simulation during preoperative consultation on PROs after breast reconstructive surgery. |
| La Padula et al. | 2019 | France | None | Retrospective cohort | Present the FFS preoperative planning of authors' male-to-female transsexual patients using photo-editing software, computer-aided design (CAD), modeling, and 3D printing |
| La Padula et al. | 2022 | Italy | None | Prospective cohort | 1) To assess the aesthetic similarity between a pre- operative 3D simulation generated using Arbrea breast simulation software and real post-operative outcomes, with a focus on patient satisfaction |
| Lekakis et al. | 2018 | Belgium | NR | Prospective cohort | 1) Comparing 2D morphing with its 3D counterpart from the patient’s and the surgeon’s perspective with a view to identifying the added value of the latter in the interest of preoperative evaluation during the rhinoplasty consult. |
| Obeid et al. | 2022 | Saudi Arabia | NR | Prospective cohort | 1) To investigate the role of simulated digital photography in rhinoplasty and whether it affects patient satisfaction. |
| Overschmidt et al. | 2018 | USA | Yes | Prospective cohort | Determine whether inclusion of three-dimensional simulation during preoperative consultation improved patient-reported outcomes and impacted mammometrics. |
| Selamioğlu et al. | 2024 | Turkey | None | Retrospective cohort | 1) To evaluate the impact of 3D imaging simulations on the decision to undergo rhinoplasty surgery. |
| Sharp et al. | 2002 | UK | NR | Retrospective cohort | 1) Measured patient satisfaction on computer imaging by means of a similar questionnaire to that employed in a previously published series  2) Analyse effects of computer imaging on overall patient benefit by comparing GBI scores on patients who had pre-operative imaging and those who did not |
| Sobral et al. | 2021 | Brazil | None | Case series | 1) To present a simple, low-cost method for 3D simulation to plan rhinoplasty. |
| Vuyk et al. | 1997 | Netherlands | NR | Retrospective cohort | 1) To document the opinion of Dutch patients on computer simulation together with the possible influence on communication and on the patient-doctor relationship 2) Compare the computer simulated prediction tracings and postoperative results by both the surgeon and by the patient |
| Yu and Jang | 2014 | South Korea | None | Retrospective cohort | 1) To evaluate the effectiveness of preoperative computer simulation (PCS) in Asian rhinoplasty patients. |

NR: not reported, UK: United Kingdom, USA: United State of America, 2D: two-dimension, 3D: three-dimension
