# Supplementary material for: Patient Perspective on Preoperative Simulation in Plastic Surgery: A Systematic Review of Patient-Reported Outcomes
Source: Plast Surg (Oakv). 2026 Aug 31:22925503261478090. Online ahead of print. doi: 10.1177/22925503261478090 (PMC13530028; doi:10.1177/22925503261478090)
Supplement: Supplementary file 2 [file sj-docx-2-psg-10.1177_22925503261478090.docx]

**Supplemental Table 2. Patient Characteristics**

| Author | Total # of Patients | # Simulation Patients | # Controls | Sex | | Mean Age (range) | Mean BMI  (range) | Time to FU (range) |
| --- | --- | --- | --- | --- | --- | --- | --- | --- |
|  |  |  |  | Male | Female | years | kg/m^2 | months |
| Costa et al. | 7 (14 breasts) | 7 (14 breasts) | NA | 0 | 7 | 35 (24-44) | 22.7 (20.4-26.5) | 0.4, 0.75, and 3 |
| Creasman et al. | 46 (92 breasts) | 46 (92 breasts) | NA | 0 | 46 | 32 | NR | 6: 35 patients  18: 28 patients |
| de Runz et al. | 38 (76 breasts) | 38 (76 breasts) | NA | 0 | 38 | 31.8 | NR | 6 |
| Donfrancesco et al. | 150 (300 breasts) | 150 (300 breasts) | NA | 0 | 150 | NR | NR | 6 |
| Godden et al. | 117 | 37 | 41: verbal description  39: 2D photographs | 0 | 117 | Total: 59  Control: 59  2D: 64  Simulated: 55 | Total: 29  Control: 28  2D: 29  Simulated: 28 | 3 and 12 |
| Hutchinson et al. | 54 | 26 | 28 | 0 | 54 | Simulated: 37.6 (24-69)  Control: 46 (26-73) | NR | NR |
| Kuroda et al. | 69 | 37 | 32 | 0 | 69 | Total: 52.7 | Total: 26.05 | 6 |
| La Padula et al. | 25 | 25 | NA | 25 | 0 | NR | NR | 6 & 12 |
| La Padula et al. | 40 (80 breasts) | 40 (80 breasts) | NA | 0 | 40 | 29.5 (18-53) | 25.8 (23-29) | 12 |
| Lekakis et al. | 172 | 172 | NA | 58 | 114 | 31.1 (15-72)  Male range: 15-29 Female range: 30-72 | NR | NR |
| Obeid et al. | 51 | 28 | 23 | 15 | 36 | Total: 27.02 (17-46) | NR | 12 |
| Overschmidt et al. | 85 | 72 | 13 | 0 | 85 | Simulated: 36.1  Control: 40.2 | Simulated: 21.8  Control: 22.6 | Simulated: 6.9  Control: 6.6 |
| Selamioğlu et al. | 146 | 146 | NA | 38 | 108 | 30.94 (18-64) | NR | NR |
| Sharp et al. | 56 | 25 | 31 | NR | NR | NR | NR | 9 (6-24) |
| Sobral et al. | 3 | 3 | NA | 2 | 1 | 23.66 (21-26) | NR | 17 (14-18) |
| Vuyk et al. | 50 (59 procedures) | 50 (59 procedures) | NA | 10 | 40 | 31 (8-65) | NR | 17 (4-36) |
| Yu and Jang | 224 | 68 | 156 | 127 | 97 | Total: 27.5 (16-61) | NR | 20.1 (13-43) |

BMI: body mass index, FU: follow-up, kg: kilogram, m: meter, NA: not applicable, NR: not reported, 2D: two-dimension, #: number
