# Supplementary material for: Patient Perspective on Preoperative Simulation in Plastic Surgery: A Systematic Review of Patient-Reported Outcomes
Source: Plast Surg (Oakv). 2026 Aug 31:22925503261478090. Online ahead of print. doi: 10.1177/22925503261478090 (PMC13530028; doi:10.1177/22925503261478090)
Supplement: Supplementary file 3 [file sj-docx-3-psg-10.1177_22925503261478090.docx]

**Supplemental Table 3. Simulation Characteristics**

| **Author** | **Surgical Procedure** | **Type of Surgery** | **Technology Used** | **Simulation Time** |
| --- | --- | --- | --- | --- |
| Costa et al. | Aesthetic Breast Surgery | Implant-based breast augmentation | Vectra M3 | NR |
| Creasman et al. | Aesthetic Breast Surgery | Implant-based breast augmentation | 4D Precision Light Breast Imaging System | NR |
| de Runz et al. | Aesthetic Breast Surgery | Implant-based breast augmentation | Crisalix | NR |
| Donfrancesco et al. | Aesthetic Breast Surgery | Implant-based breast augmentation | Vectra + Precision Light Software | NR |
| Godden et al. | Oncologic Breast Surgery | Breast conserving surgery | Vectra XT + Mirror Software | NR |
| Hutchinson et al. | Aesthetic Breast Surgery | Implant-based breast augmentation | Vectra | NR |
| Kuroda et al. | Oncologic Breast Surgery | Breast conserving surgery + bilateral mammaplasty: 45  Mastectomy + implant-based reconstruction: 24 | Crisalix | NR |
| La Padula et al. | Rhinoplasty | Aesthetic rhinoplasty | Morpheus Photo  Warper® photoediting software  Zortrax® 3D printer | Mean 145 +/- 13.2 minutes |
| La Padula et al. | Aesthetic Breast Surgery | Implant-based breast augmentation | Arbrea Breast Software | 90 seconds |
| Lekakis et al. | Rhinoplasty | Aesthetic: 109 (63%)  Functional-Aesthetic: 63 (37%)  Open: 109 (63%)  Closed: 63 (37%) | 2D Photoshop CS3 Extended Version 10 & Vectra H1 | 2D: 3-5 minutes  3D: 10-15 minutes |
| Obeid et al. | Rhinoplasty | Aesthetic | Vectra H1 | NR |
| Overschmidt et al. | Aesthetic Breast Surgery | Implant-based breast augmentation | Vectra | NR |
| Selamioğlu et al. | Rhinoplasty | Aesthetic | Vectra | NR |
| Sharp et al. | Rhinoplasty | Aesthetic: 9  Functional: 18  Both: 29 | Compaq notebook computer with a Pentium 3 processor, RGB flat screen monitor & Nikon Coolpix 950 (105.mm) digital camera | NR |
| Sobral et al. | Rhinoplasty | Aesthetic: 1  Functional-Aesthetic: 2 | RhinOnBlender | 60 minutes |
| Vuyk et al. | Craniofacial Surgery | Otoplasty: 2  Rhinoplasty: 47  Chin augmentation and rhinoplasty: 9  Chin augmentation: 1 | Computer Imaging (IBM compatible personal computer, flat screen RGB monitor, CCD videocamera with RGB and digital output, summagraphics 850 noteboard)  AT + T true  vision image processing software was used for manipulation ofthevideoimage | NR |
| Yu and Jang | Rhinoplasty | increase tip projection (n = 45; 66.2%)  increase rotation (n = 19; 27.9%)  narrow a wide bulbous tip (n = 10; 14.7%)  correct asymmetry (n = 7; 10.3%).  Dorsal deformities included  flat nose (n = 33; 48.5%)  deviated nose (n =25; 36.8%)  hump (n = 22; 32.4%)  cleft lip (n = 2; 2.9%).  68/68 open rhinoplasty | Canon D7 Camera + Syncromax version 3.0; DreamWizard, Seoul, South Korea | NR |

NR: not reported
