# Supplementary material for: Patient Perspective on Preoperative Simulation in Plastic Surgery: A Systematic Review of Patient-Reported Outcomes
Source: Plast Surg (Oakv). 2026 Aug 31:22925503261478090. Online ahead of print. doi: 10.1177/22925503261478090 (PMC13530028; doi:10.1177/22925503261478090)
Supplement: Supplementary file 4 [file sj-docx-4-psg-10.1177_22925503261478090.docx]

**Supplemental Table S4.**

Table A.

RoB2 (Risk of Bias Tool – Version 2) – Assessment of Randomized Controlled Studies

| Study | Risk of bias from randomization | Risk of bias due to deviations from intended interventions | Risk of bias due to missing outcome data | Risk of bias in measurement of the outcome | Risk of bias in selection of the reported result | Overall risk of bias |
| --- | --- | --- | --- | --- | --- | --- |
| Godden et al. (2021) | Low risk | Some concerns | Low risk | Low risk | Low risk | Low risk |
| Kuroda et al. (2024) | Low risk | Some concerns | Some concerns | Some concerns | Low risk | Low risk |

Table B.

MINORs (Methodological Index for Non-Randomized Studies) Evaluation – For Comparative Studies (Maximum Score: 24)

| Study | Clearly stated aim | Inclusion of consecutive patients | Prospective collection of data | Endpoints appropriate to the aim of the study | Unbiased assessment of study endpoint | Follow-up period appropriate to aim of study | Loss to follow-up <5% | Prospective calculation of study size | Adequate control group | Contemporary groups | Baseline equivalence of groups | Adequate statistical analysis | Total |
| --- | --- | --- | --- | --- | --- | --- | --- | --- | --- | --- | --- | --- | --- |
| Hutchinson et al. (2021) | 2 | 0 | 0 | 2 | 1 | 1 | 1 | 0 | 1 | 0 | 2 | 2 | 10 |
| Lekakis et al. (2019) | 2 | 2 | 2 | 2 | 1 | 2 | 2 | 0 | 2 | 2 | 2 | 2 | 17 |
| Obeid et al. (2022) | 2 | 0 | 2 | 2 | 1 | 2 | 2 | 0 | 1 | 2 | 1 | 2 | 17 |
| Overschmidt et al. (2018) | 2 | 0 | 2 | 2 | 1 | 2 | 1 | 0 | 1 | 2 | 1 | 2 | 16 |
| Sharp et al. (2002) | 2 | 2 | 0 | 2 | 1 | 2 | 1 | 0 | 1 | 2 | 1 | 2 | 16 |
| Vuyk et al. (1997) | 2 | 2 | 0 | 2 | 1 | 2 | 0 | 0 | 2 | 2 | 0 | 2 | 15 |
| Yu and Jang (2014) | 2 | 2 | 0 | 2 | 1 | 2 | 2 | 0 | 2 | 2 | 1 | 2 | 18 |

Table C.

MINORs (Methodological Index for Non-Randomized Studies) Evaluation – For Non-Comparative Studies (Maximum Score: 16)

| Study | Clearly stated aim | Inclusion of consecutive patients | Prospective collection of data | Endpoints appropriate to the aim of the study | Unbiased assessment of study endpoint | Follow-up period appropriate to aim of study | Loss to follow-up <5% | Prospective calculation of study size | Total |
| --- | --- | --- | --- | --- | --- | --- | --- | --- | --- |
| Costa et al. (2017) | 2 | 2 | 2 | 2 | 1 | 1 | 2 | 0 | 12 |
| Creasman et al. (2011) | 2 | 2 | 2 | 2 | 1 | 2 | 1 | 0 | 12 |
| De Runz et al. (2017) | 2 | 2 | 0 | 2 | 1 | 2 | 1 | 2 | 12 |
| Donfrancesco et al. (2013) | 2 | 2 | 1 | 2 | 2 | 2 | 2 | 0 | 13 |
| La Padula et al. (2019) | 2 | 0 | 0 | 2 | 1 | 2 | 2 | 0 | 9 |
| La Padula et al. (2022) | 2 | 0 | 2 | 2 | 1 | 2 | 2 | 0 | 11 |
| Selamioğlu et al. (2024) | 2 | 0 | 0 | 2 | 1 | 2 | 2 | 0 | 9 |
| Sobral et al. (2021) | 2 | 2 | 2 | 2 | 1 | 2 | 2 | 0 | 13 |
